# Striated Muscle as Implantation Site for Transplanted Pancreatic Islets

**DOI:** 10.1155/2011/352043

**Published:** 2011-12-07

**Authors:** Daniel Espes, Olof Eriksson, Joey Lau, Per-Ola Carlsson

**Affiliations:** ^1^Department of Medical Cell Biology, Husargatan 3, P.O. Box 571, Uppsala University, 75123 Uppsala, Sweden; ^2^Preclinical PET Platform, Department of Medicinal Chemistry, Uppsala University, 75123 Uppsala, Sweden; ^3^Department of Medical Sciences, Uppsala University, 75185 Uppsala, Sweden

## Abstract

Islet transplantation is an attractive treatment for selected patients with brittle type 1 diabetes. In the clinical setting, intraportal transplantation predominates. However, due to extensive early islet cell death, the quantity of islets needed to restore glucose homeostasis requires in general a minimum of two donors. Moreover, the deterioration of islet function over time results in few insulin-independent patients after five-year followup. Specific obstacles to the success of islet transplantation include site-specific concerns for the liver such as the instant blood mediated inflammatory reaction, islet lipotoxicity, low oxygen tension, and poor revascularization, impediments that have led to the developing interest for alternative implantation sites over recent years. Within preclinical settings, several alternative sites have now been investigated and proven favorable in various aspects. Muscle is considered a very promising site and has physiologically properties and technical advantages that could make it optimal for islet transplantation.

## 1. Introduction

Type 1 diabetes mellitus is a chronic disease with typical onset during childhood or adolescence. Patients suffering from the disease require multiple daily insulin injections coordinated alongside vigilant monitoring of blood glucose levels. For some patients, glucose homeostasis is not optimized despite these efforts, leading to repeated hyperglycemic and hypoglycemic events. Such repeated episodes of severe hypoglycemia, requiring medical assistance, predicate a patient being considered for islet transplantation. Currently, intraportal transplantation is performed in the majority of clinical cases. The liver was selected as the optimal implantation site for islet transplantation in the beginning of the 1970s, the basis of experimental studies by Kemp et al. [1]. However, clinical results of islet transplantation were meager with less than 10% of transplant recipients displaying insulin independency. The introduction of the Edmonton-protocol ten years ago drastically improved the outcome to a rate of 80% insulin independency after one year [2]. Nevertheless, this procedure requires the islets of multiple donors and provides inadequate long-term results. Only 10% of transplant recipients have maintained insulin-independency after five years despite continuing efforts [3]. Fundamentally, however, intraportal transplantation must be considered successful. The main stimulus for islet transplantation, repeat episodes of severe hypoglycemia, is prevented in most patients irrespective of insulin independence after transplantation and results in a marked increase in the quality of life.

 During recent years, interest in alternative implantation sites with the potential to improve long-term results has produced advances in noninvasive methodologies towards the quantification of islet graft mass and function. Positron emission tomography (PET) is one such technique, providing a functional imaging modality that quantifies an in vivo distribution of bioactive compounds labeled with positron-emitting nuclides. Commonly, clinically used PET tracers are labeled with short-lived radionuclides that result in a radiation dose that is both localized and nontoxic, allowing for multiple scans of a single patient.

## 2. Obstacles with Intraportal Islet Transplantation

The rapid islet cell death observed in intraportal transplantation is in part caused by an instant blood-mediated inflammatory response (IBMIR) [4, 5]. The IBMIR consists of both thrombotic and complement activation cascades which lead to the formation of blood clots around the islets, leucocyte infiltration, and disruption of islet morphology [6]. In clinical settings, the IBMIR can be detected immediately after islet infusion as an increase in both thrombin-antithrombin complexes and increased c-peptide levels due to beta-cell disruption and death [5]. With a combined positron emission tomography and computed tomography (PET/CT) technique, the early cell death after intraportal islet transplantation has been estimated at approximately 25% [7].

In conjunction to the IBMIR, characteristic hypoxic events have also been contributed to early islet cell death. Indeed, during the process of initial islet isolation, the native vascular connections are disrupted, enforcing the islets to depend on oxygen diffusion immediately after transplantation. This process is further complicated when considering that the liver parenchyma has a much lower oxygen tension than can be found in the pancreas [8]. We recently observed in an experimental animal model that approximately 70% of islets are hypoxic one day after intraportal transplantation [9]. This finding was accomplished employing pimonidazole, a biochemical marker that accumulates in islet cells with oxygen tension levels below 7.5–10 mmHg. Notably, the hypoxia may even be underestimated in these experiments, as pimonidazole adducts are not incorporated within dead or dying cells. In addition, our team has also observed that caspase-3 staining of islets reveals an apoptosis level, correlating to the hypoxia, as high as 10% in islets one day after intraportal transplantation [10].

It has been proposed that the high concentrations of immunosuppressive drugs in the portal vein, following intestinal uptake of the drugs, may have a toxic effect on transplanted islet cells in the liver [11, 12]. Moreover, several long-term liver-site-specific challenges have been identified such as lipotoxicity to the transplanted cells [13, 14] and insufficient vascular engraftment [15, 16]. Intrahepatically transplanted islets are slowly revascularized, severely impairing blood perfusion and oxygenation up to three months after transplantation [17]. Considering the aforementioned challenges, substantial hurdles remain to be overcome to improve the long-term prognosis of existing intraportal islet transplantation procedures.

## 3. Considerations for a New Implantation Site

Over the last decade, there has been an increased interest in alternative implantation sites due to the identified obstacles and the deterioration of islet graft function after time following intraportal transplantation. Several factors must be considered when choosing an optimal implantation site. The prospective organ requires physiological characteristics that are favorable for islets in regards to oxygen tension, vascular supply, and angiogenesis. Implanted beta cells must also have the capacity to adapt to the implantation organ and maintain their differentiation. Furthermore, a viable route for insulin secretion and glycemic detection is required. Some sites, such as the testis, thymus, brain, and eye are immune-privileged making them tempting candidates. Considering the technical and surgical aspects, an ideal site should have easy access, allowing for a minimally invasive procedure. Preferably, it should be possible to obtain biopsies without causing undue risk to either the patient or the graft. There is current optimism towards developing methods able to quantify the islet cell mass with imaging techniques. Several PET probes intended for noninvasive visualization of endogenous beta cells in the pancreas have been investigated during recent years, generally targeting the monoamine pathways as these are absent in exocrine tissue which compose the vast majority (>95%) of the pancreatic volume. PET tracers have been utilized for studies of the central nervous system (CNS) since the 1980s, in particular for the serotonergic and dopaminergic systems, although various tracers have now been applied to image endogenous beta cells. These include carbon-11, fluorine-18, or gallium-68 labeled versions of small molecule ligands or peptides, targeting vesicular monoamine transport [18] and oxidation as well as serotonin and dopamine synthesis [19, 20]. Other recently available PET tracers act as ligands for beta cell-specific markers such as sulfonylurea receptor-1 [21] and glucagon-like peptide-1 receptor [22]. It has also been suggested that early beta cell destruction due to autoimmunity or rejection can be studied by [^18^F]FDG, a marker for glucose metabolism, imaging both inflammatory lesions and insulitis [23]. Technical advances which image endogenous beta cell mass can naturally be applied in islet transplantation studies.

The pancreas is a tempting implantation site. It is the natural milieu for islets. In experimental studies we have shown that the vascular density in islets transplanted to the pancreas is only slightly lower than in endogenous islets [24]. We have also shown that in contrast to intrahepatically transplanted islets, islets transplanted to the pancreas display less severe changes in gene expression and have only moderate changes in their metabolic function posttransplantation [25]. However, injection into the pancreas may elicit leakage of exocrine enzymes, resulting in pancreatitis. Therefore, the pancreas has rarely been considered as a clinically feasible implantation site. Improved implantation techniques, for example, beneath the pancreatic capsule, as well as safety and efficacy studies in large animal models are clearly needed before considering clinical trials.

Superior islet survival has recently been demonstrated following experimental transplantation into the oment when compared to intraportal islet transplantation [26]. Since the 1980s, the omental pouch has been considered as a potential site for islet transplantation [27]. A primate model study revealed an observable delay in transplant engraftment when compared to intraportal transplantation, yet over time, recipients obtained equitable plasma C-peptide levels [28]. Although the vascular engraftment of islets in oment is delayed, the omentum or omental pouch has the capacity to be a useful clinical site.

The anterior chamber of the eye provides excellent conditions for engraftment of transplanted islets [29]. Injected islets engraft on the iris become fully revascularized and have the capacity to reverse diabetes in mice [29]. A recent follow-up study in diabetic baboons also showed successful long-term survival and function of islets after allogeneic transplantation [30]. Furthermore, transplanted islets contributed to glycemic control without any perceived impairment of vision. The implantation procedure was minimally invasive, and considering the immune-privileged properties of this site, it can be concluded as an attractive site for clinical use. However, pertinent safety and efficacy studies remain, bearing in mind the potential impact on transplant recipients visual function if transplanting islets of a sufficient magnitude to reverse hyperglycemia.

## 4. Intramuscular Islet Transplantation: A Promising Implantation Site

Striated muscle has been used for decades as a site for autologous transplantation of normal parathyroid tissue in patients with hyperparathyroidism, with excellent results [31]. Such grafts have proven highly functional for more than 10 years, with full restoration of calcium homeostasis [31]. The procedure is well documented with few complications and is considered a standard technique.

Muscle has a naturally occurring angiogenesis with a higher oxygen tension than can be found in the liver parenchyma. We have recently shown that there is a rapid revascularization of intramuscularly transplanted mouse and human islets, containing a blood vessel incidence on par with native islets within two weeks posttransplantation [32]. Graft blood perfusion was restored and the oxygen tension of intramuscular islets only slightly diminished compared to native islets after revascularization [33]. This paradigm is further supported in the clinical setting and witnessed through magnetic resonance imaging of pancreatectomized patients receiving autotransplantation of isolated islets to the brachioradialis muscle, where a high revascularization of intramuscular islet grafts has also been observed [32]. Experimentally, islets transplanted to muscle also have a superior glucose tolerance compared to recipients of similar numbers of islets transplanted intraportally [32]. A case report from the Nordic Network for Clinical Islet Transplantation, where a young patient with hereditary pancreatitis underwent total pancreatectomy treated by an autologous islet transplant to the brachioradialis muscle, also showed sustained insulin production from the graft for more than two years [34].

**Figure 1:**
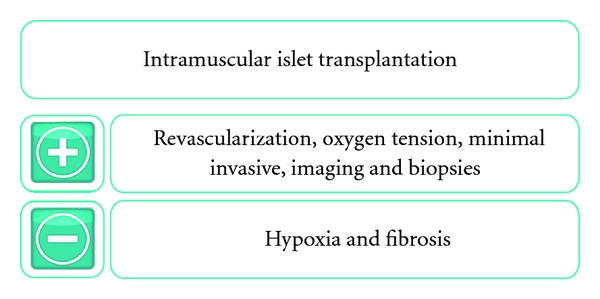
Summary of beneficial aspects and known obstacles for intramuscular islet transplantation.

However, it is arguably the implantation technique itself that is pivotal to a high success rate at the intramuscular site. Injection of large clusters of islets causes substantial early islet cell death due to hypoxia. In such grafts, massive fibrosis can be observed both experimentally and clinically [32, 33] (Figure 1). This could explain the previous erratic success in experimental and clinical intramuscular islet transplantation [35–37]. It had become apparent to our group that in order to improve early islet survival, a shift in the transplantation procedure was required that disperses the islets throughout the tissue, for example, transplanting the islets along a “pearls on a string” fashion. Early cell death might be further reduced by remodeling the transplant site with bioengineered matrices and/or cotransplantation of oxygen carriers such as perfluorocarbons. There is also the possibility of improving the vascular network in the muscle prior to transplantation, since higher vascular density could easily be induced by angiogenic stimulators or hypoxia, both of which have a strong stimulatory effect on angiogenesis in muscle [38].

Due to minimal islet-blood contact within intramuscular transplantation, the challenge presented by the IBMIR is reduced. The capacity to transplant islets into a designated area in the muscle also facilitates site remodeling of immunological events prior to transplantation. Specifically, this could include modulation of early inflammation with mesenchymal stem cells, providing the potential to accelerate revascularization, as shown in both intraportal islet transplantation [39, 40] and islets transplanted beneath the kidney capsule [41]. Mesenchymal stem cells could either be cotransplanted or injected in advance to modulate the site.

It has earlier been reported that even moderate exercise causes hypoglycemia in rats with islets transplanted to the liver, kidney, and peritoneal cavity [42]. Hypoglycemic events can also be observed following intrapancreatic transplantation of islets [43]. Upregulation of lactate dehydrogenase in transplanted islets, with concomitantly increased lactate production eliciting insulin release during periods of low blood glucose levels, may be an explanation for this [25, 44]. It has not yet been reported how lactate dehydrogenase levels and other gene expression are affected in betacells of intramuscular islet grafts. Exercise might also cause a site-specific challenge to intramuscular islet grafts due to intramuscular pressure changes, since the glucose-consumption of muscle cells increases during exercise in accord with the regional blood flow.

## 5. Monitoring of Islets at the Intramuscular Site

It is difficult to obtain biopsies of islets transplanted into the liver. Islets are spread throughout the liver parenchyma, and liver biopsies *per se* are not free of risk. However, in the muscle, a biopsy would be simple to obtain. Preemptively dividing the transplanted islet mass, designating a specific islet mass to a separate site intended for potential biopsies, would allow subsequent studies to identify early markers for rejection.

The feasibility of imaging transplanted islets through PET techniques depends to a large extent on the site of implantation. This can be understood by examining the composition of a PET tracer signal emitted within a given region in vivo. In the main, a signal containing a composite of specific binding is produced (i), or the PET probe bound to the receptor of interest is proportional to receptor density. Displaying nonspecific binding (ii), the PET probe may also bind to other structures, revealing vascular contribution (iii). Vascular contribution is vital, as the tissue uptake of tracer is limited by local perfusion, potentiating an underestimation of uptake in grafts with low revascularization. All of these factors influence our ability to study islet grafts in different tissues.

With these issues in mind, we can see that longitudinal noninvasive visualization of hepatic grafts presents a considerable challenge, with substantial graft dilution (i). In addition, it is difficult to design PET tracers with a low hepatic background signal. Most tracers are metabolized in the liver (ii). The perfusion (iii), however, is sufficient to transport the tracer to engrafted islets in hepatic sinusoids shortly after transplantation.

This paradigm is reversed when considering intramuscular islet grafts. Grafts are generally pure and concentrated (i), with the above-mentioned PET tracers effecting a low to negligible uptake in muscle tissue (ii). Revascularization is therefore the limiting factor for visualization of the graft. It has recently been shown that intramuscular islet grafts attain a vascularization comparable to that in pancreatic islets after approximately 2 weeks [32]. Considering these illustrations, quantification of intramuscular islet grafts containing an adequate volume is potentially achievable after engraftment and revascularization has been completed.

Progress with visualizing has been made both in preclinical and clinical studies of intrahepatic and intramuscularly transplanted islets using PET. The hepatic distribution and survival of islets during the peritransplant phase, following intraportal islet transplantation, has been studied with ex vivo labeling of islets by [^18^F]FDG prior to infusion in murine [45] and porcine [46] models as well as in the clinic [7]. However, longitudinal studies are not yet possible using the ex vivo labeling methodology.

Uptake in an intramuscular islet graft of a vesicular monoamine transporter 2-(VMAT2-) ligand, [^11^C]DTBZ, correlated well to the observable decrease in blood sugar of a preclinical STZ mouse model in a study by Witkowski et al. [47]. However, interpretation of the results and translation to the clinical situation is problematic when considering that the islets were implanted using a bioscaffold.

Pattou et al. [48] showed proof-of-principle in the clinic by visualizing islets transplanted to the brachioradialis muscle in a type 1 diabetic patient by intravenous administration of a [^111^In]-labeled exendin-4, a GLP-1R ligand, using SPECT, an imaging modality related to PET.

**Figure 2:**
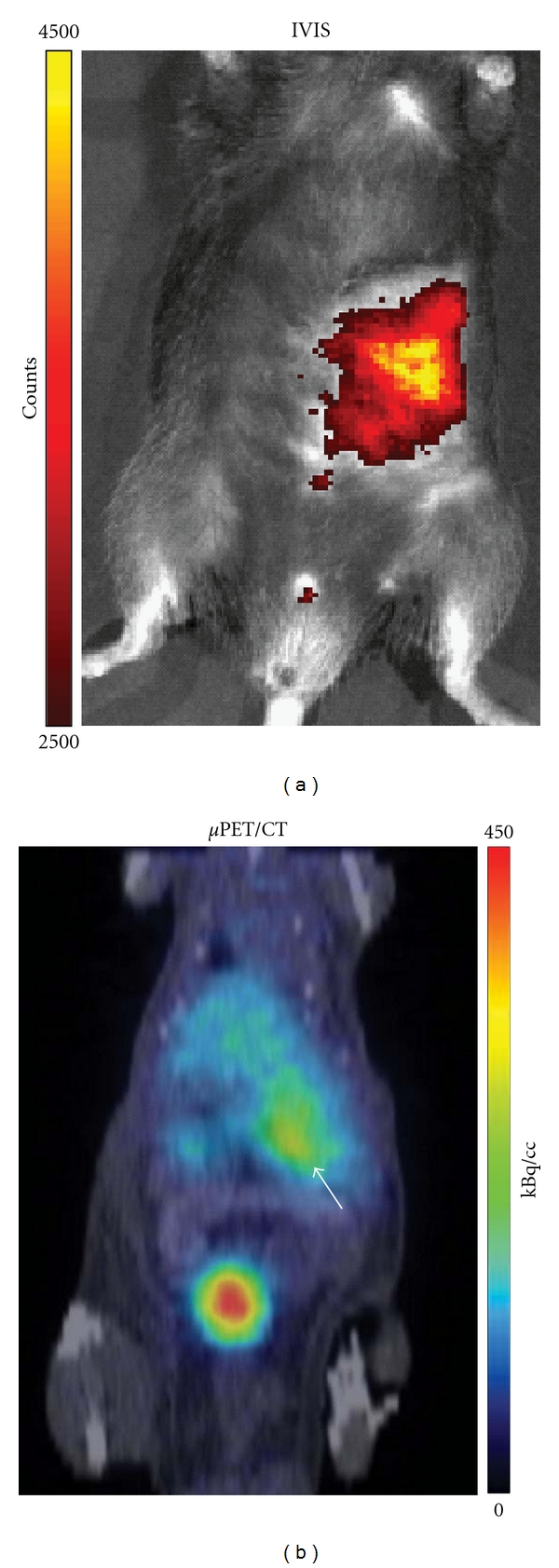
PET imaging of intramuscularly transplanted islets using the VMAT2 ligand [^18^F]FE-DTBZ-d4 as PET tracer. An inbred C57BL/6 mouse was transplanted with 300 mouse islets to the left abdominal muscle. The islets were labeled with Q-tracker prior to implantation. One month later, the mouse was administered the PET tracer [^18^F]FE-DTBZ-d4 intravenously. The tracer uptake could be detected by a *μ*PET/CT scanner after 60 minutes (right image, orange delineation). The corresponding location of the islets as determined by Q-tracker fluorescence emission is shown to the left.

The VMAT2 ligand [^18^F]FE-DTBZ-d_4_ (see Figure 2) and the catecholamine precursor [^18^F]L-DOPA (unpublished data) are currently being investigated as biomarkers for transplanted beta cells in two ongoing preclinical studies of intramuscularly transplanted islets in mice.

## 6. Conclusions

Striated muscle is a promising implantation site and in many regards superior to the liver. However, it is not yet fully characterized regarding islet long-term survival and functionality. The obstacles with early islet cell death observed to date following intramuscular islet transplantation can likely be overcome through remodeling the implantation site. Further studies capable of actualizing effective strategies are clearly needed, within both small and large animal models.
